# High V‐PPase activity is beneficial under high salt loads, but detrimental without salinity

**DOI:** 10.1111/nph.15280

**Published:** 2018-06-25

**Authors:** Dorothea Graus, Kai R. Konrad, Felix Bemm, Meliha Görkem Patir Nebioglu, Christian Lorey, Kerstin Duscha, Tilman Güthoff, Johannes Herrmann, Ali Ferjani, Tracey Ann Cuin, M. Rob G. Roelfsema, Karin Schumacher, H. Ekkehard Neuhaus, Irene Marten, Rainer Hedrich

**Affiliations:** ^1^ Institute for Molecular Plant Physiology and Biophysics University of Würzburg Julius von‐Sachs Platz 2 Würzburg D‐97082 Germany; ^2^ Institute of Bioinformatics Center for Computational and Theoretical, Biology University of Würzburg Am Hubland Würzburg D‐97218 Germany; ^3^ Centre for Organismal Studies Developmental Biology of Plants Ruprecht‐Karls‐University of Heidelberg Im Neuenheimer Feld 230 Heidelberg 69120 Germany; ^4^ Plant Physiology University Kaiserslautern Postfach 3049 Kaiserslautern D‐67653 Germany; ^5^ Department of Biology Tokyo Gakugei University Nukui Kitamachi 4‐1‐1 Koganei‐shi Tokyo 184‐8501 Japan; ^6^ Tasmanian Institute of Agriculture University of Tasmania Hobart TAS 7001 Australia

**Keywords:** cell death, plasma membrane voltage, proton pump currents, salt, vacuolar pH, vacuolar proton‐ATPase (V‐ATPase), vacuolar proton‐pyrophosphatase (V‐PPase)

## Abstract

- The membrane‐bound proton‐pumping pyrophosphatase (V‐PPase), together with the V‐type H^+^‐ATPase, generates the proton motive force that drives vacuolar membrane solute transport. Transgenic plants constitutively overexpressing V‐PPases were shown to have improved salinity tolerance, but the relative impact of increasing PP_i_ hydrolysis and proton‐pumping functions has yet to be dissected.
- For a better understanding of the molecular processes underlying V‐PPase‐dependent salt tolerance, we transiently overexpressed the pyrophosphate‐driven proton pump (NbVHP) in *Nicotiana benthamiana* leaves and studied its functional properties in relation to salt treatment by primarily using patch‐clamp, impalement electrodes and pH imaging.
- NbVHP overexpression led to higher vacuolar proton currents and vacuolar acidification. After 3 d in salt‐untreated conditions, V‐PPase‐overexpressing leaves showed a drop in photosynthetic capacity, plasma membrane depolarization and eventual leaf necrosis. Salt, however, rescued NbVHP‐hyperactive cells from cell death. Furthermore, a salt‐induced rise in V‐PPase but not of V‐ATPase pump currents was detected in nontransformed plants.
- The results indicate that under normal growth conditions, plants need to regulate the V‐PPase pump activity to avoid hyperactivity and its negative feedback on cell viability. Nonetheless, V‐PPase proton pump function becomes increasingly important under salt stress for generating the pH gradient necessary for vacuolar proton‐coupled Na^+^ sequestration.

## Introduction

Membrane‐bound proton‐pumping pyrophosphatases utilize inorganic Mg_2_PP_i_ as an energy‐rich compound to drive the uphill transport of protons from the cytosol into the vacuolar lumen. According to the amino acid sequence and K^+^ dependency, H^+^‐PPases can be subdivided into two groups. The type‐I H^+^‐PPase (designated here as V‐PPase) together with the V‐ATPases are localized in the vacuolar membrane, while the K^+^‐independent type‐II H^+^‐PPase resides in the Golgi and leads to the acidification of the Golgi lumen upon proton pumping (Segami *et al*., 2010). Direct patch‐clamp measurements established that the ATP and PP_i_‐powered proton pumps localize in the same type of vacuole and work in concert (Hedrich *et al*., 1989; Krebs *et al*., 2010). Consequently, a vacuole membrane voltage of *c*. –30 mV and a pH gradient of usually *c*. 2 pH units are generated (Krebs *et al*., 2010; Shen *et al*., 2013; Wang *et al*., 2015). These gradients energize the voltage‐dependent and proton‐driven ion and metabolite transporters (De Angeli *et al*., 2006; Schulz *et al*., 2011; Klemens *et al*., 2014).

The heteromeric V‐ATPase consists of 13 different subunits, which assemble to the peripheral ATP‐hydrolyzing V_1_ domain and the membrane‐integral, proton‐translocating V_0_ domain (Schumacher & Krebs, 2010). In comparison, the V‐PPase is formed by a homodimer of a single polypeptide with a molecular mass of *c*. 80 kDa. The primary structure of V‐PPases was first ascertained from *Arabidopsis thaliana* (Sarafian *et al*., 1992), followed by many other land plants such as barley and mung bean. These revealed a high degree of conservation in the amino acid sequences with an identity of 86–91% (Maeshima, 2000). Three genes encoding for membrane‐bound H^+^‐PPases (*AtVHP1‐3*) were identified in *A. thaliana*, with the type‐I H^+^‐PPase AtVHP1 as the only one targeted to the vacuolar membrane (AtVHP1, also referred to as AVP1 in the literature; Sarafian *et al*., 1992; Segami *et al*., 2010). Other species such as *Nicotiana tabacum* and *Oryza sativa* appear to contain three and even six V‐PPase isoforms, respectively (Lerchl *et al*., 1995; Sakakibara *et al*., 1996; Choura & Rebai, 2005; Liu *et al*., 2010). Based on the crystal structure and mutants generated from the V‐PPase of mung bean, proton translocation conveyed by this electroenzyme is proposed to take place along a pathway formed by protonable amino acid residues and two bound water molecules (Lin *et al*., 2012).

In earlier studies, it was shown that under nonstressed conditions, the loss of V‐ATPase function is not compensated by up‐regulation of V‐PPase pump activity. This defect caused an increase in vacuolar pH from 5.9 to 6.4, resulting in a retarded growth phenotype (Krebs *et al*., 2010). In plants lacking both V‐ATPases and V‐PPase, the mesophyll vacuole proton concentration becomes even lower and the accompanying growth retardation is more pronounced (Kriegel *et al*., 2015). However, the sole lack of V‐PPase function only leads to a mild growth phenotype, which can be rescued by expression of a soluble pyrophosphatase (Ferjani *et al*., 2011). Thus, the V‐PPase appears to have dual cellular functions: regulation of cytosolic PP_i_ homoeostasis and the generation of a membrane electrochemical proton gradient, with the latter contributing to the proton motive force (PMF) that drives secondary active transport processes.

Under salt stress, the PMF across the vacuole membrane is used by NHX‐type proton‐coupled Na^+^‐K^+^ transporters for vacuole sequestration of cytotoxic Na^+^ overloads (Apse *et al*., 2003; Yamaguchi *et al*., 2003; reviewed in Yamaguchi *et al*., 2013; and controversially discussed in Jiang *et al*., 2010). Accordingly, plants should benefit from a higher trans‐tonoplast proton gradient. This would enhance cytosolic Na^+^ detoxification and so increase salt tolerance. In agreement with this notion, stable overexpression of V‐PPases in tobacco, cotton and poplar plants leads to improved performance under salt stress (Gao *et al*., 2006; Duan *et al*., 2007; Pasapula *et al*., 2011; Yang *et al*., 2015). Nonetheless, the possibility that improved stress tolerance results from pleiotropic responses and adaptations to overexpression of V‐PPases during plant development cannot be excluded. To eliminate such indirect effects, we transiently overexpressed the pyrophosphate‐driven proton pump and studied the action of V‐PPase under PMF‐consuming salt conditions.

## Materials and Methods

### Plant material and growth conditions

*Nicotiana benthamiana* was cultivated on soil with a day : night regime of 14 : 10 h, 26 : 22°C, and a photon flux density of 300 μmol m^−2^ s^−1^. Humidity was *c*. 60% in the glasshouse. Experiments were carried out on 4‐ to 6‐wk‐old plants.

### Reconstruction of V‐PPase amino acid sequences from *N. benthamiana*

*Nicotiana benthamiana* V‐PPases were identified using a *de novo* assembled transcriptome, profile‐based open reading frame annotations and a conditional reciprocal Best Blast search. Raw RNA‐seq paired‐end reads (SRR685298, SRR696884, SRR696915, SRR696938, SRR696940, SRR696961, SRR696988, SRR696992, SRR697013) were downloaded from the NCBI Sequence Read Archive. Reads were trimmed using Skewer (v.0.1.99, ‐Q 30 ‐q 30 ‐l 60 ‐m pe, 10.1186/1471‐2105‐15‐182) and assembled with Trinity (Release 2013‐02‐25, –jaccard_clip –group_pairs_distance 500 –path_reinforcement_distance 75 –min_kmer_cov 2; Grabherr *et al*., 2011). Resulting transcripts (241 752 isoforms; 125 729 unigenes) were annotated using TransDecoder. Putative V‐PPases were identified by searching with the Pfam profile PF03030 against all annotated open reading frames using HMMSearch (v.3.0; Eddy, 2011). Possible *N. benthamiana* V‐PPases homologous to AtVHP1 were identified by performing a conditional reciprocal Best Blast search against the annotated protein set of *A. thaliana* (Tair10; Lamesch *et al*., 2012).

### Transient overexpression of soluble and vacuolar pyrophosphatases

For transient overexpression of different pyrophosphatases (NbVHP1, NbVHP2, AtVHP1, IPP1 from *Saccharomyces cerevisiae*; Ferjani *et al*., 2011) in *N. benthamiana* mesophyll cells, the complementary DNA was cloned under the ubiquitin promoter into the pCambia2200 vector using the USER‐System (Nour‐Eldin *et al*., 2006). This vector additionally carried the coding sequence for free enhanced green fluorescent protein (eGFP; Zhang *et al*., 1996) under the control of the double CaMV35S promoter (CaMV, cauliflower mosaic virus) (Kay *et al*., 1987). When any pyrophosphatase cDNA was inserted, the GFP‐containing vector was used as a control. Tobacco leaves were transformed using the agroinfiltration method, essentially as described by Jung *et al*. (2015) and Te *et al*. (2005), with the agromix solution containing 10 mM MgCl_2_, 10 mM MES (pH 5.6/KOH), 150 μM acetosyringone. For additional salt treatment, the agrobacterium suspension was supplemented with 200 mM NaCl and infiltrated into *N. benthamiana* leaves. The leaf surface was not contaminated with the agro‐mix suspension during agroinfiltration.

### Isolation of mesophyll protoplasts

Mesophyll protoplasts were enzymatically isolated essentially as described by Beyhl *et al*. (2009). The enzyme solution was adjusted to an osmolality of 500 mOsmol kg^−1^ with d‐sorbitol. After enzyme incubation for 60 min, the protoplast suspension was filtered through a nylon mesh (50 μm) and washed with 500 mM d‐sorbitol plus 1 mM CaCl_2_. Protoplasts were collected by centrifugation (7 min at 60 ***g*** and 4°C) and removal of the supernatant.

### Detection of GFP fluorescence

Fluorescent images were taken from mesophyll protoplasts and vacuoles with a confocal laser scanning microscope (TCS SP5; Leica, Wetzlar, Germany. The plant material was excited with an argon laser at 488 nm to detect the GFP fluorescence emission between 505 and 530 nm and for the autofluorescence of chloroplasts, between 580 and 620 nm using the acousto‐optical beam splitter of the SP5.

For patch‐clamp experiments, the vacuoles of transiently transformed mesophyll cells were identified in the recording chamber by the emission of GFP fluorescence from the vacuole‐attached fluorophores. After excitation at 470 nm with a precisExcite High‐Power LED Illumination system (Visitron System, Puchheim, Germany) mounted on an Axiovert A1 microscope (Zeiss, Oberkochen, Germany), GFP fluorescence was detected with an emission filter between 480 and 535 nm (Chroma Technology Corp., Bellows Falls, VT, USA).

### RNA isolation and quantitative real‐time PCR

After isolation and enrichment, *N. benthamiana* wild‐type mesophyll protoplasts were immediately frozen in liquid nitrogen and stored at −80°C until use. Total RNA of protoplasts was obtained using dynabeads^®^ (Invitrogen Dynal AS, Oslo, Norway). Afterwards, first‐strand cDNA was generated from 2.5 μg RNA using M‐MLV reverse transcriptase (Promega, Fitchburg, MA, USA) according to the manufacturer's recommendation. Quantitative real‐time polymerase chain reaction (pPCR) was carried out with Realplex^®^ (Eppendorf, Hamburg, Germany), using the Absolute SYBR Capillary mix (Thermo Fisher Scientific, Waltham, MA, USA). Transcription data were normalized to 10 000 molecules of actin using standard curves calculated for the individual PCR products. The following primers were used: NbVHP1fwd (5′‐GTT GGA ATC TTG TTT GGC‐3′) and NbVHP1rev (5′‐GTA GGA GTG GTA TTC GTT‐3′), NbVHP2fwd (5′‐CCT CAT AGT TGG GAT TTT C‐3′) and NbVHP2rev (5′‐GAA TGC CTC CCA ACA AGC TC‐3′), NbACTfwd (5′‐CCC AGA AGT CCT CTT‐3′) and NbACTrev (5′‐GGG ATG CGA GGA T‐3′).

### pH imaging of mesophyll vacuoles

Two days after infiltration and 2–3 h before vacuolar pH imaging, leaves were infiltrated with 200 μM BCECF‐AM diluted in 10 mM MgCl_2_, 10 mM MES pH 5.6 (KOH) and kept in the dark at room temperature until measurement. Either agrobacteria with a pCambia3300‐based binary vector harboring a UBQ10 promotor and NbVHP1 coding sequence was coinfiltrated, or an equivalent amount of 19K agrobacteria strain (White & Cipriani, 1990; Te *et al*., 2005) was infiltrated as a control. Vacuolar pH imaging of mesophyll cells was performed on an inverted Zeiss microscope AxioObserver (Zeiss) equipped with a Plan‐Apochromat 20×/0.8 NA objective. Leaf disks were glued with the abaxial side to the cover slip using Medical Adhesive (VM 355; Ulrich AG, St Gallen, Switzerland) and were flooded with medium containing 10 mM MgCl_2_ and 10 mM MES, pH 5.6 (KOH). Illumination was performed with a high‐speed polychromator system (Visitron Systems, Puchheim, Germany) controlled by VisiView software, and fluorescence was captured with a 512 × 512 pixel Evolve EMCCD camera (Photometrics, Tucson, AZ, USA). For BCECF‐AM, excitation wavelengths of 440 nm or 500 ± 5 nm were used. Fluorescence emission was reflected via a dual‐band dichroic mirror 440/520, filtered with a dual‐band emitter 464/547 (Semrock Inc., Rochester, NY, USA) and ET 535/30 nm bandpass filter (Chroma Technology Corp., Bellows Falls, VT, USA). Ten leaves with or without NbVHP1 expression under nonsalt‐ and salt‐treated conditions were imaged, and > 100 regions were analyzed in each leaf. Image processing was performed using the public domain NIH ImageJ program v.1.51 (developed at the US National Institutes of Health and available on the Internet at http://rsb.info.nih.gov/nih-image/).

### Chlorophyll fluorescence measurements

Plants were dark‐adapted for 30 min before the Chl fluorescence of detached leaves was monitored with a Maxi PAM fluorometer (AVT 033), recorded and analyzed with Imaging Win v.2.41a FW MULTI RGB (Walz, Effeltrich, Germany). The minimum fluorescence of photosystem II (*F*_0_) and the maximum fluorescence of dark‐adapted cells (*F*_m_) were measured to calculate the maximum photochemical quantum yields of photosystem II ($F_{v}/F_{m}$) according to the equation 

$$F_{v}/F_{m}=\left(F_{m}-F_{0}\right)/F_{m}.$$

### Patch‐clamp experiments

Two to three days after agroinfiltration, mesophyll protoplasts were isolated and stored on ice until aliquots were taken to liberate vacuoles for patch‐clamp experiments. Release of vacuoles was achieved by incubation of protoplasts for 8 min in hypotonic medium composed of 10 mM EGTA, 10 mM HEPES/Tris pH 7, adjusted to 270 mOsmol kg^−1^ with d‐sorbitol. Patch‐clamp experiments on vacuoles were performed in the whole‐vacuole configuration, essentially as described elsewhere (Beyhl *et al*., 2009; Schulz *et al*., 2011). Patch pipettes were prepared from Kimax‐51 melting point borosilicate glass capillaries (Kimble of DWK Life Science, Wertheim, Germany) or GB150T‐8P glass capillaries (Science Products, Hofheim, Germany) and had a pipette resistance of 3–4 MΩ under standard solute conditions. Macroscopic currents were recorded at a data acquisition rate of 20 ms and low‐pass‐filtered at 100 Hz with an EPC‐8 patch‐clamp amplifier (HEKA, Elektronik Lambrecht, Germany). The data were digitized with an ITC‐16 computer interface (Instrutech Corp., Elmont, NY, USA). Data acquisition and offline analysis were performed using the software programs Pulse (HEKA Electronik, Lambrecht, Germany) and IgorPro (Wave Metrics Inc., Lake Oswego, OR, USA). Macroscopic currents were measured at a holding voltage of 0 mV under symmetrical solute conditions and normalized to the compensated membrane capacitance (*I* *C*_m_^−1^) as a measure for the vacuolar size. The standard pipette and bath solution, facing the luminal and cytosolic sides of the vacuolar membrane, respectively, was composed of 100 mM K‐gluconate, 0.1 mM CaCl_2_, 5 mM MgCl_2_, 10 mM Hepes/Tris, pH 7.5. When the pipette solution was adjusted to pH 5.5, 10 mM Mes/Tris was used instead of HEPES/Tris. The osmolality of the solutions was adjusted to 500 mOsmol kg^−1^ with d‐sorbitol. Unless otherwise noted, pyrophosphate (K_4_‐PP_i_) and ATP (Mg^2+^ salt) were applied at concentrations of 150 μM and 5 mM, respectively, to the cytosolic side of the vacuolar membrane by employing a manual application system as described by Schulz *et al*. (2011). Apart from this energy‐rich substance, the composition of the application pipette solution was identical to the bath medium. Except during ATP or PP_i_ application, the bath solution was continuously perfused, so enabling constant ionic conditions in the bath chamber, and washout of ATP or PP_i_ after administration. Any changes in the standard solute conditions are detailed in the figure legends.

### Measurements of membrane voltage *in planta*

Square‐shaped sections taken from leaves were mounted with their abaxial side up into a Petri dish with double‐sided adhesive tape, flooded with bath solution (5 mM KCl, 1 mM CaCl_2_, 10 mM MES, pH 6) and placed on an Axioskop 2FS microscope (Zeiss). The microelectrodes (double‐barreled) and the reference electrode were prepared as described by Voss *et al*. (2016). A piezo‐driven micromanipulator (MM3A, Kleindiek Nanotechnik, Reutlingen, Germany) was used to drive the microelectrode into the cell, and the membrane potential was recorded with a CA‐100 amplifier equipped with HS‐180 headstages (BioLogic, Seyssinet‐Pariset, France).

### Sequence data

Sequence data from this article can be found in the European Nucleotide (ENA) archive under the EBI ENA accession numbers LT883169 (*NbVHP1*), LT883170 (*NbVHP2*).

## Results

### Transient V‐PPase overexpression accelerates pyrophosphate‐driven vacuole proton currents

Although ion pumps are present in relatively high copy numbers compared with ion channels, the turnover numbers of these metabolic energy‐fueled machines is only in the order of 10–10^3^ ions s^−1^, so they produce only weak electrical currents (Hedrich, 2012; Lodish *et al*., 2012). However, fairly substantial current densities are required for the high‐resolution recordings needed for detailed characterization of the V‐PPase pump function. Hence, to increase current resolution, we boosted the number of V‐PPase pump proteins in the vacuolar membrane by exploiting an overexpression approach. Since in our hands Arabidopsis lines constitutively overexpressing AtAVP1 showed no significant increase in PP_i_‐induced vacuolar currents, we took advantage of *N. benthamiana* transient transformation (Latz *et al*., 2007; De Angeli *et al*., 2013). For transformation using the agroinfiltration method, a plasmid was generated that allowed the identification of transformed vacuoles by virtue of a GFP fluorescence signal. To prevent unintended effects on the pump activity (Segami *et al*., 2014), we did not use a translational GFP fusion to this pump protein. Instead, the coding sequences of AtVHP1 and the GFP were separately but tandemly expressed under the control of ubiquitin and the double CaMV35S promoter, respectively (Supporting Information Fig. S1a). As a control, we used a construct containing just the GFP. After infiltration of tobacco leaves with an *A. tumefaciens* suspension carrying one of these plasmids, the vacuoles released from transformed mesophyll cells were identified from the GFP fluorescence signal emitted from vacuole‐attached fluorophores (Fig. S2a). Only fluorescent vacuoles were used for patch‐clamp experiments. To record the proton pump activity of the total vacuolar V‐PPase pump population, the whole‐vacuole patch‐clamp configuration was established. After equilibration of the vacuolar sap with the medium in the patch pipette, membrane currents were measured at a membrane voltage of 0 mV under symmetrical solute and stable pH condition. These conditions avoid interference with background ion fluxes driven by any voltage and/or proton gradient (Schulz *et al*., 2011). In analogy to our earlier electrophysiological studies on the V‐ATPase (Rienmüller *et al*., 2012), the V‐PPase proton pump activity was triggered by cytosolic administration of the energy‐rich pyrophosphate moiety. Upon application of 150 μM pyrophosphate to the cytosolic side of the vacuole membrane, an upward deflection of the current amplitude was recorded (Fig. S2b). After removal of the pump substrate, the current amplitude returned to the original current baseline level, that is, that measured before PP_i_ stimulation. With respect to our experimental conditions, these PP_i_‐induced outward currents reflect proton pumping into the vacuolar lumen, driven by the energy from PP_i_ hydrolysis. Under standard solute conditions, vacuoles transformed with AtVHP1 in addition to GFP, exhibited proton pump current responses that were three times higher than currents from control vacuoles released from mesophyll cells expressing only GFP (Fig. S2c). When the PP_i_‐induced current responses were recorded from the same vacuole in the presence of either 10 mM EGTA or 1 mM CaCl_2_ at the cytosolic side of the vacuolar membrane, a threefold difference was observed in the pump currents for both AtVHP1‐harboring vacuoles and control vacuoles, being always higher under CaCl_2_‐free conditions (Fig. S2d). Thus, in the presence of elevated cytosolic Ca^2+^ concentrations, the Ca_2_PP_i_ rather than the Mg_2_PP_i_ complex is most likely formed. This prevents the electroenzymes from properly hydrolyzing PP_i_. Consequently, less energy from PP_i_ hydrolysis is then available for proton pumping. This inhibitory Ca^2+^ effect on AtVHP1, as well as on the endogenous NbVHP‐mediated proton pump currents, corresponds with the Ca^2+^ dependency reported earlier for the hydrolytic enzyme activity of AtVHP1 and the mung bean V‐PPase (VrVHP) (Maeshima, 1991; Drozdowicz *et al*., 2000), and the proton pump activity of VrVHP (Nakanishi *et al*., 2003).

### *Nicotiana benthamiana* vacuoles functionally express two V‐PPase isoforms

**Figure 1:**
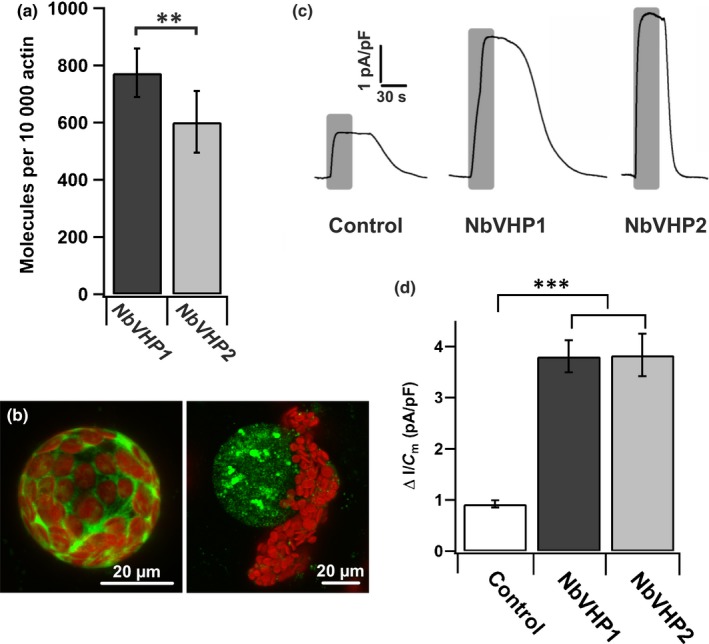
Expression and function of NbVHPs in *Nicotiana benthamiana* mesophyll cells. (a) Transcript abundances of *NbVHP1* and *NbVHP2* in mesophyll protoplasts isolated from noninfiltrated leaves. *n* = 4 independent experiments, means ± SE; **, *P *<* *0.01, Student's *t*‐test. (b) Confocal fluorescence images of a protoplast (left) and vacuole (right) released from different mesophyll cells overexpressing NbVHP1 together with free green fluorescent protein (GFP). Red fluorescence is a result of chloroplast autofluorescence. Bars, 20 μm. (c) Representative pyrophosphate‐induced current responses of vacuoles released from mesophyll cells overexpressing free GFP together with either NbVHP1 or NbVHP2 after agroinfiltration. As a control, GFP was overexpressed alone. Duration of pyrophosphate treatment (150 μM) is indicated by the superimposed gray bars. Total number of experiments with individual vacuoles is given in (d). (d) Maximal pyrophosphate‐induced changes in current density of vacuoles released from mesophyll cells overexpressing free GFP alone (control, *n* = 10) or free GFP together with either NbVHP1 (*n* = 14) or NbVHP2 (*n* = 4) after agroinfiltration. Pyrophosphate was applied at a concentration of 150 μM. Data points represent means ± SE. Asterisks indicate significant differences compared with the control (Student's *t*‐test): ***, *P *<* *0.001.

Although the Arabidopsis and tobacco V‐PPases share a similar function, proton currents in one vacuole produced by a mixed population of pumps from both species do not allow unequivocal conclusions to be made. Taking into account the proton pump current responses of control vacuoles, one‐third of the PP_i_‐induced pump current responses from AtVHP1‐harboring vacuoles probably corresponds to the activity of endogenous V‐PPases from *N. benthamiana* (Fig. S2b,c). In order to replace AtVHP1 with the native V‐PPase for transient overexpression in tobacco, we identified the molecular nature of NbVHP. The *N. benthamiana* genome contains eight putative V‐PPase genes, scattered over a highly fragmented assembly. These genes differ in only a few coding sites but have ambiguous gene flanking regions, which strongly point towards assembly artefacts or highly polymorphic regions/introns. Reliable transcripts were directly reconstructed from RNA‐seq data using Trinity (Haas *et al*., 2013). This analysis procedure avoids the generation of possible pseudogenes as well as highly polymorphic regions in introns and gene‐flanking genomic regions. *AtVHP1* was compared with the assembled *N. benthamiana* transcriptome, and only two transcripts were found to contain a full‐length cDNA of a V‐PPase, which we named *NbVHP1* (EBI ENA accession LT883169) and *NbVHP2* (EBI ENA accession LT883170). To test whether both *NbVHP* genes are expressed in tobacco leaves, qRT‐PCR experiments were performed on cDNA generated from isolated mRNA of mesophyll protoplasts. Both *NbVHP1* and *NbVHP2* were found to be expressed in mesophyll cells in comparable amounts (Fig. 1a). The amino acid sequence alignments between NbVHP1 and NbVHP2 displayed a high sequence identity of 91% to each other and 88–91% with AtVHP1 from *A. thaliana* and VrVHP from *Vigna radiata* (Fig. S3a,b). Moreover, these V‐PPases share all the conserved amino acid residues that have a proposed function in substrate binding (Mg_2_PP_i_, K^+^, Mg^2+^), hydrolysis of Mg_2_PP_i_ and H^+^ translocation (Fig. S3a). Thus, NbVHP1 and NbVHP2 are likely to represent the functional V‐PPases of *N. benthamiana*. To test this assumption and to directly quantify the PP_i_‐dependent pump activity, currents were analyzed in patch‐clamp experiments. As described for *AtVHP1*,* NbVHP1* and *NbVHP2* were cloned in plasmids for agrobacterium‐mediated transformation of *N. benthamiana* leaves (Fig. S1a). Again, expression of the NbVHPs was indirectly displayed by visualization of GFP fluorescence (Fig. 1b). When the V‐PPase pump activity of vacuoles from transformed mesophyll cells was measured in response to 150 μM PP_i_ (Fig. 1c,d), the proton pump current density was almost four times higher in vacuoles from NbVHP1‐ or NbVHP2‐overexpressing mesophyll cells than in the control vacuoles (Fig. 1d).

We also tested whether, in this tobacco system, overexpressed NbVHPs are only targeted to the vacuolar membrane or if some pump proteins might also be mistargeted to the plasma membrane (Segami *et al*., 2014). Thus, we also overexpressed an mGFP‐labeled NbVHP1 construct in tobacco leaves (Fig. S1b; Methods S1). When the plasma membrane of a mesophyll protoplast was stained with the red fluorescent dye FM4‐64, the mGFP signal did not colocalize with the FM4‐64 signal, but instead was only detected in the vacuolar membrane (Fig. S4). Therefore, these results indicate that NbVHP1 is exclusively targeted to the vacuolar membrane and both NbVHPs represent functional V‐PPases in tobacco leaves. Given that we measured comparable transcript numbers for *NbVHP1* and *NbVHP2* (Fig. 1a), it can be assumed that the background PP_i_‐induced pump currents recorded in control vacuoles are generated by the shared activities of NbVHP1 and NbVHP2. Consequently, when either NbVHP1 or NbVHP2 is overexpressed, the recorded PP_i_‐induced proton pump currents and deduced electrical characteristics are primarily related to the overexpressed V‐PPase pump protein type under investigation (Fig. 1d).

### Overexpression of V‐PPases leads to cell death

**Figure 2:**
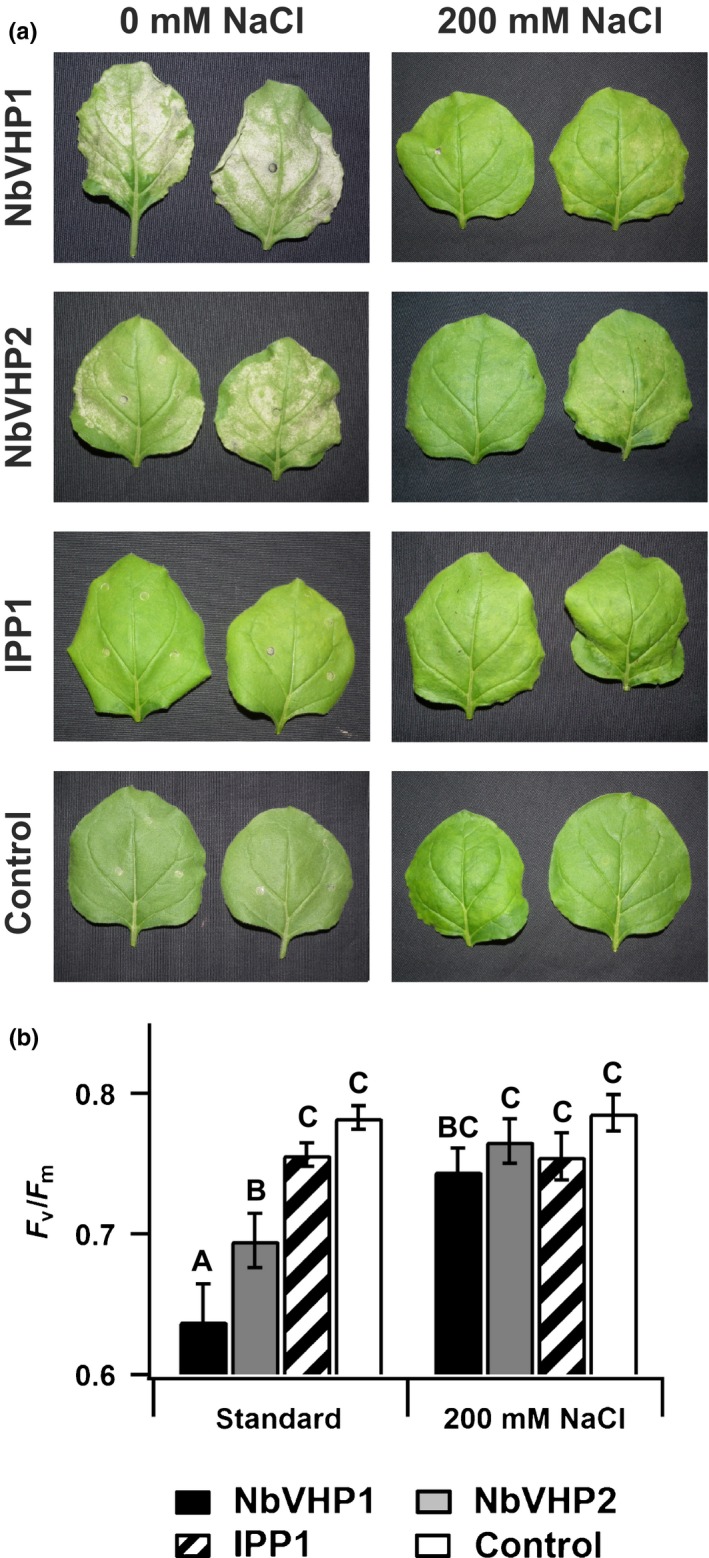
Salt stress rescues V‐PPase‐overloaded *Nicotiana benthamiana* leaves from cell death. (a) Images from leaves overexpressing free green fluorescent protein (GFP) together with either NbVHP1 or NbVHP2 at 3 d after agroinfiltration with or without 200 mM NaCl. (b) Maximum photochemical quantum yields of photosystem II (*F*_v_/*F*_m_) determined from detached leaves overexpressing free GFP alone (control) or free GFP together with the indicated pyrophosphatases at 3 d after agroinfiltration with or without 200 mM NaCl. Number of independent experiments is *n* = 6 (means ± SE). Significant differences between values at *P *<* *0.05 (Duncan test), determined by statistical analysis with the software Sas 9.0 (http://www.sas.com/), are indicated by different letters.

Surprisingly, when the performance of transformed leaves was monitored over a longer period, pronounced necrosis was observed. This always developed 3 d after agroinfiltration of each of the V‐PPase tested (NbVHP1, NbVHP2, AtAVP1), but not with GFP alone (Figs 2a, S2e). In addition, the necrotic V‐PPase‐dependent effect was accompanied by an impaired photosynthetic activity (Fig. 2b). It has been shown that the V‐PPase plays a role not only in generating a membrane electrochemical proton gradient, but also in cytosolic PP_i_ homoeostasis (Ferjani *et al*., 2011). To further distinguish between the impact of these two V‐PPase functions for cell viability, *N. benthamiana* leaves were infiltrated with a soluble PPase, in addition to either *NbVHP1* or *NbVHP2*. The soluble PPase IPP1 from *S. cerevisiae* was chosen because of its ability to complement the loss of AtVHP1 function in the *fugu5‐1* mutant in PP_i_ homoeostasis (Ferjani *et al*., 2011). In our studies, IPP1‐transformed *N. benthamiana* leaves exhibited a hydrolytic PP_i_ activity that was *c*. 10‐fold higher than in control vacuoles. This indicates the functional expression of IPP1 (Fig. S5; Methods S2). However, in contrast to the V‐PPase overloaded leaves, neither was leaf necrosis observed nor was the photosynthetic capacity impaired in IPP1‐overexpressing plants (Fig. 2). This implies that the fourfold higher proton‐pump activity (Fig. 1d) in the V‐PPase‐overexpressing leaves does impair cellular pH homoeostasis.

### Salt treatment rescues the V‐PPase‐overloaded leaves from cell death

The increased demand for proton‐coupled solute transport across the vacuolar membrane, such as during vacuolar H^+^‐coupled Na^+^ sequestration under saline conditions, should counteract the negative cellular impact of an increased V‐PPase proton‐pump activity. Therefore, leaves were infiltrated with 200 mM NaCl. As a result, the leaf Na^+^ content increased from five‐ to 10‐fold compared with nonstressed conditions, then remained almost stable for over 2 d regardless of whether leaves were simultaneously agroinfiltrated with free GFP (control) or different types of pyrophosphatase (Fig. S6a,b; Methods S3). Despite the increased total Na^+^ leaf content, 3–5 h after infiltration of the hyperosmotic NaCl solution (425 mOsmol kg^−1^), the osmolality of the extracted leaf apoplast fluid was similar to control leaves (Fig. S6c; Methods S4). Thus, the infiltrated salt did not remain in the leaf apoplast but was rapidly taken up into the leaf cells where it would be directed into the vacuole to prevent detrimental cytotoxic effects. Na^+^ and Cl^−^ are probably directed to the vacuole via a proton‐coupled transport mechanism that takes advantage of the prevailing pH gradient across the tonoplast. This view is strongly supported by the finding that under saline conditions, V‐PPase‐overexpressing leaves did not exhibit any necrosis and retained their full photosynthetic activity (Fig. 2). Moreover, GFP‐ and IPP1‐overexpressing leaves did not suffer from salt treatment, as indicated by the absence of necrotic leaf tissue alongside a sustained high photosynthetic capacity (Fig. 2).

### Salt overloads increased expression and proton pumping of V‐PPases

**Figure 3:**
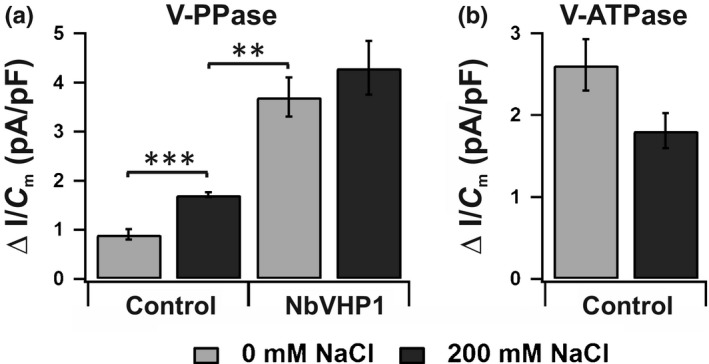
Effect of salt treatment on pump activity of V‐PPase and V‐ATPase. (a) Maximal V‐PPase‐mediated proton pump current responses of vacuoles from *Nicotiana benthamiana* mesophyll protoplasts overexpressing free green fluorescent protein (GFP) alone or together with NbVHP1 for 2 d. Agroinfiltration was carried out with or without 200 mM NaCl. Pump activity of V‐PPase (*n* = 4–10; **, *P *<* *0.01; ***, *P *<* *0.001, Student's *t*‐test) was triggered by cytosolic application of 150 μM pyrophosphate. (b) Maximal V‐ATPase‐mediated proton pump current responses of vacuoles from *N. benthamiana* mesophyll protoplasts (*n* = 7; *P* = 0.059, Student's *t*‐test), overexpressing free GFP at 2 d after agroinfiltration in the presence or absence of 200 mM NaCl. For studies of the V‐ATPase pump activity, the Ca^2+^ concentration in the bath and pipette medium was increased to 1 mM and Kgluconate was replaced by KCl. The pump activity was triggered by cytosolic application of 5 mM ATP. Data points represent means ± SE.

Cytosolic extrusion of Na^+^ into the vacuole by NHX‐type transporters is linked to the proton gradient across the tonoplast, which is generated by V‐ATPases together with V‐PPases. To test the impact of V‐PPase‐related proton pumping in salt management in tobacco, the salt effect on the expression level and the proton pump activity of the NbVHPs was examined. Two days after salt infiltration alone, the transcript abundance of the endogenous *NbVHP1* and *NbVHP2* were strongly increased when compared with salt‐free conditions (Fig. S6d; Methods S5). In line with the increased endogenous *NbVHP* expression level under salt conditions (Fig. S6d), a twofold rise in PP_i_‐induced pump currents was monitored in mesophyll vacuoles from salt‐treated leaves compared with control leaves (Fig. 3a). However, these endogenous V‐PPase‐mediated proton pump currents recorded under salt conditions were still less than half of the proton pump currents measured in vacuoles from *NbVHP1*‐overexpressing mesophyll protoplasts (Fig. 3a). With the latter vacuoles, salt treatment did not cause any further significant rise in PP_i_‐driven proton pump currents. Hence, the *NbVHP1*‐transformed mesophyll cells appear to have fully exploited their capacity to produce V‐PPase pump proteins. When the salt effect on the endogenous V‐ATPase pump activity of control vacuoles was also examined, it turned out that the V‐ATPase‐mediated proton pump currents were decreased by *c*. 31% (Fig. 3b). Therefore, in contrast to normal growth conditions, the higher V‐PPase proton pump activity under salinity compensated at least partially for the reduction in V‐ATPase‐dependent proton pumping in tobacco mesophyll cells.

### Changes in vacuolar pH do not feed back on NbVHP activity

**Figure 4:**
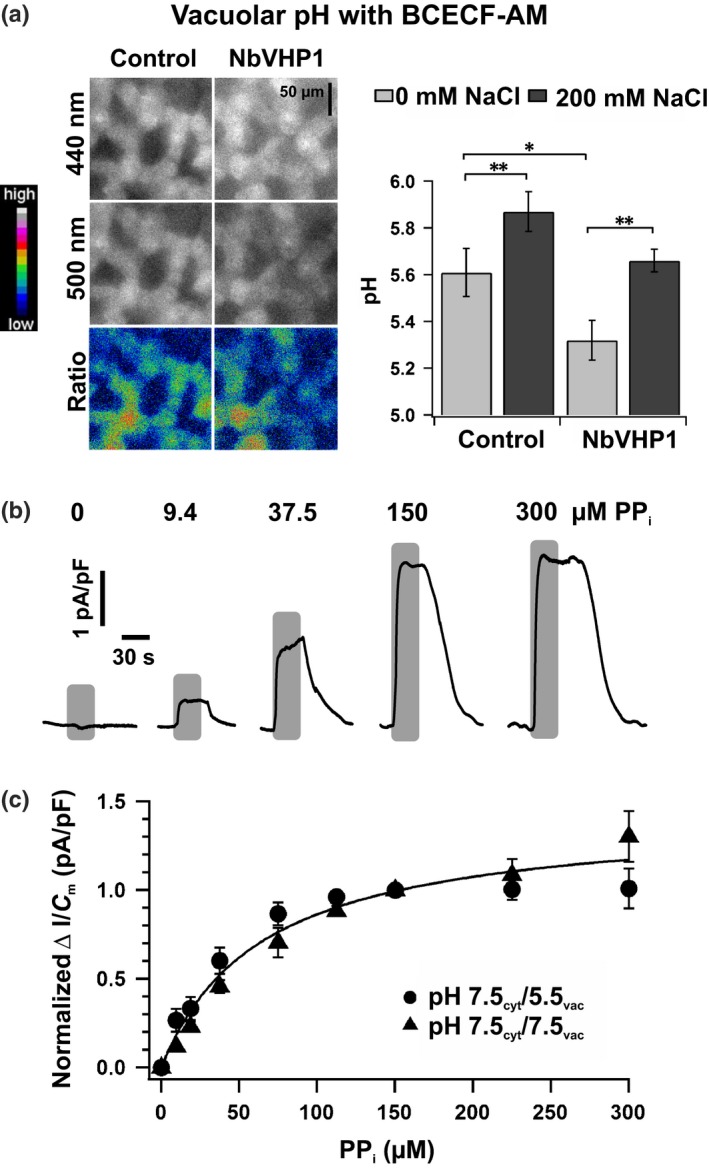
Dependence of V‐PPase‐mediated proton pump currents on pyrophosphate concentration and luminal pH. (a) Vacuolar pH of *Nicotiana benthamiana* mesophyll cells, transformed with or without NbVHP1 in the absence or presence of 200 mM NaCl, was visualized using BCECF‐AM (*n* = 10). Images in the upper panel were taken from salt‐untreated leaves. The fluorescence ratio is shown in pseudocolor images and the emission at the corresponding excitation wavelength is presented in grayscale. Quantification of the vacuolar pH ratio value with (dark gray) or without (light gray) 200 mM NaCl infiltration is displayed (cf. Supporting Information Fig. S8 and Methods S6 for pH calibration with BCECF). Data are means ± SE (*, *P *<* *0.05; **, *P *<* *0.01, Student's *t*‐test). (b) Representative current responses of one *N. benthamiana* mesophyll vacuole to the application of a range of pyrophosphate concentrations as indicated above the current traces. Using symmetrical solute conditions with pH 7.5 at both sides of the vacuolar membrane, the experiment was conducted with a vacuole released from an NbVHP1/GFP‐overexpressing mesophyll cell. Duration of pyrophosphate treatment is indicated by the superimposed gray bars. (c) Maximal pyrophosphate‐induced changes in current density of vacuoles released from NbVHP1/GFP‐overexpressing *N. benthamiana* mesophyll cells, plotted against the pyrophosphate concentration. Current responses were normalized to those recorded from the same vacuole during the application of 150 μM pyrophosphate. Experiments were performed at a luminal pH of 7.5 (triangles, *n* = 4–10) or pH 5.5 (circles, *n* = 3–5). Data points (means ± SE) were globally fitted with a Michaelis–Menten equation (solid line).

To test whether the rise in the proton pump activity is large enough to cause a drop in the vacuolar pH, NbVHP1‐overexpressing mesophyll cells of *N. benthamiana* were infiltrated with the membrane‐permeable, pH‐sensitive fluorescent dye BCECF‐AM (Fig. 4a). For this particular experiment, we used a vector construct that expressed only NbVHP1, thus avoiding spectral overlap of GFP and BCECF. The fluorescence BCECF‐ratio signals associated with *NbVHP1*‐overexpressing cells revealed a significant decrease in the vacuolar pH from 5.6 to 5.3 compared with control tissue under nonsaline conditions. By contrast, when the leaf tissue was treated with salt, the vacuolar pH of control mesophyll cells significantly increased from pH 5.6 to 5.9. Moreover, the vacuolar pH of NbVHP1‐overloaded mesophyll cells changed significantly, by *c*. 0.3 pH units, rising from pH 5.3 to the pH level of control cells under nonsaline conditions (Fig. 4a).

To elucidate whether changes in the vacuolar pH feed back on the V‐PPase pump activity, vacuoles overexpressing NbVHP1 were challenged with different bath (cytosol) PP_i_ and luminal proton concentrations. Using symmetrical pH 7.5 conditions at both sides of the vacuolar membrane, increasing PP_i_ concentrations up to 300 μM were applied (Fig. 4b). Pump currents increased with substrate concentration, saturating above 150 μM PP_i_ (Fig. 4b,c). Similar results were obtained under a physiological pH gradient of 2 pH units (pH_cyt_ 7.5/pH_vac_ 5.5) (Fig. 4c). As variations in pump currents between vacuoles could result from different levels of NbVHP1 expression, pump currents were normalized to those recorded in 150 μM PP_i_ and plotted against the applied PP_i_ dose (Fig. 4c). In addition, owing to the pH independency of the V‐PPase‐mediated pump currents, data points of both pH conditions were assessed together and globally fitted with a Michaelis–Menten function. This revealed a *K*_m_ value of 65 ± 9 μM. When analogous experiments were conducted with vacuoles from AtVHP1‐overexpressing mesophyll cells, a similar saturation‐type dependency on the PP_i_ concentration with a *K*_m_ value of 75 ± 11 μM was determined (Fig. S2f). Although two‐thirds of the current responses of these vacuoles most probably corresponds to the activity of AtVHP1 and one‐third to the endogenous NbVHP1/2 (Fig. S2d), the *K*_m_ value did not differ from that determined from the pump current responses of NbVHP1‐overexpressing vacuoles (Fig. 4c). Thus, in line with the high homology in the primary structure, the AtVHP1 and NbVHP1 proton pumps are characterized by a similar substrate PP_i_ affinity, response to cytosolic Ca^2+^ and independence of the luminal pH. With respect to the latter feature, a drop in the luminal pH caused by a higher pump activity would not counteract the V‐PPase‐dependent proton transport capacity.

### Depolarization of the plasma membrane potential in NbVHP1‐overexpressing leaf epidermal cells

**Figure 5:**
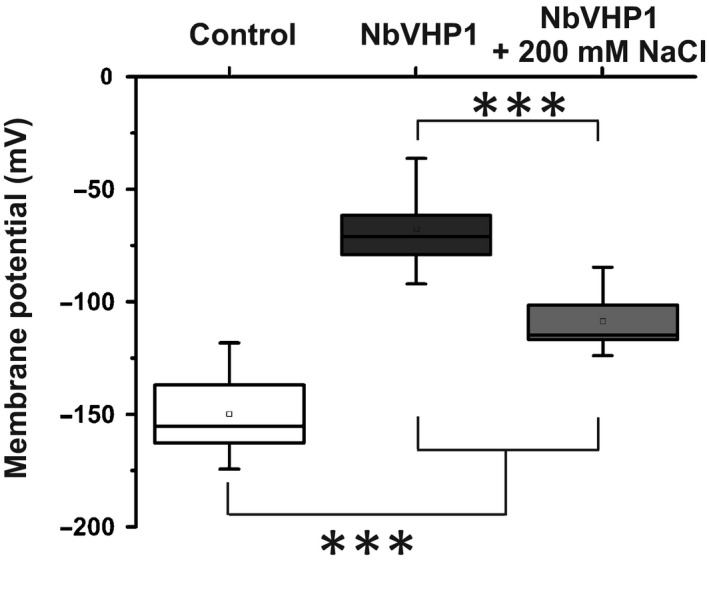
Effect of V‐PPase overexpression on membrane voltage control. Membrane voltages of *Nicotiana benthamiana* epidermal leaf cells expressing free green fluorescent protein (GFP) without (control) or together with NbVHP1 (***, *P* < 0.001, Student's *t*‐test). Measurements (*n* = 6–10) were performed 2–3 d after infiltration of transformed agrobacteria ± 200 mM NaCl. The horizontal line in the box represents the median while the upper and lower whiskers give the maximum and minimum values. Additionally, the mean value is indicated by a square.

Next, we studied the effect of NbVHP1 overexpression on membrane voltage control of epidermal leaf cells using intracellular microelectrodes. First, the subcellular localization of the electrode tips was tested by current injection of Lucifer Yellow via one barrel of a double‐barreled electrode (Wang *et al*., 2015). In all cells tested (*n* = 9), the fluorescent dye was loaded into the cytosol, indicating that the tip of virtually all impaled microelectrodes ended up in this compartment. Consequently, impaled microelectrodes were used to measure the plasma membrane voltage of *N. benthamiana* epidermal leaf cells. These recordings revealed that GFP‐overexpressing control leaves had hyperpolarized membrane voltages of *c*. –150 mV. However, 2–3 d after infiltration but before necrotic areas had developed, overexpression of NbVHP1 caused a depolarization of the plasma membrane to –85 mV (Fig. 5). This NbVHP1 overexpression‐dependent depolarization was suppressed in leaves when they were exposed to NaCl. Thus, the NbVHP overexpression phenotype indicates that the proton pump activity of the V‐PPases feeds back on the plasma membrane voltage. In the latter scenario, hyperacidification of the vacuole, which negatively feeds back to chloroplast performance and cell membrane potential, is rescued by presence of salt and proton‐coupled pumping of Na^+^ out of the cytoplasm.

## Discussion

### Transport features of NbVHPs ensure high proton transport capacity

We identified two genes (*NbVHP1*,* NbVHP2*) in *N. benthamiana* that encode for functional membrane**‐**bound, proton‐pumping V‐PPases (Fig. 1c,d). The genes share 91% identity in amino acid sequence (Fig. S3) and have comparable transcript numbers in mesophyll protoplasts (Fig. 1a). Both NbVHPs contribute to the electrochemical proton gradient across the vacuolar membrane in the leaf mesophyll cell. When one of the two NbVHPs or the Arabidopsis AtVHP1 was overexpressed in *N. benthamiana* mesophyll cells, the pump density increased several‐fold, thereby allowing high‐resolution PP_i_‐driven proton current recordings (Figs 1c,d, 4b,c, S2b–d, S7). For NbVHP1 and AtVHP1, we directly measured a PP_i_‐*K*_m_ value of *c*. 70 μM, which is in agreement with the physiological PP_i_ content of plant cells (Rea & Sanders, 1987; Weiner *et al*., 1987; Jelitto *et al*., 1992). Likewise, the maximal proton pump currents were reached within the physiological concentration range of PP_i_. Even with a two‐ to fourfold enlarged V‐PPase population, as naturally obtained under salt conditions or artificially as a result of transient overexpression, the cytosolic pool of PP_i_ is still sufficiently high to supply the pump proteins with their increased demand for the energy‐rich substrate. This notion is supported by our findings that NbVHP overexpression led to an increased PMF, which negatively impacted cell viability. Furthermore, the PP_i_ affinity of the V‐PPases of *N. benthamiana* and *A. thaliana* (NbVHP1, AtVHP1) was in the same order of magnitude, irrespective of the luminal pH; it did not feed back on PP_i_ hydrolysis of the V‐PPases. The V‐PPases share this feature of substrate affinity with the V‐ATPase residing in the same vacuole (Rienmüller *et al*., 2012). Thus, these transport features ensure that the V‐PPase is likely to operate at full capacity, particularly under stress conditions, and is not restricted by luminal pH changes or deficiency of the energy‐rich substrate.

### Individual roles of V‐PPase and V‐ATPase in salt stress management

Many reports have already explored plant responses to salt stress at the level of V‐ATPases and V‐PPases. This is because these vacuolar proton‐pumping electroenzymes can establish the PMF at the tonoplast that is required for vacuolar Na^+^ sequestration by a proton‐coupled antiporter. With respect to the V‐ATPase, the hydrolytic activity is often enhanced in response to saline treatments (Nakamura *et al*., 1992; Wang *et al*., 2001; Kabała & Kłobus, 2008; Queirós *et al*., 2009). However, as Wang *et al*. (2001) and Queirós *et al*. (2009) outlined, no common pattern in the regulation of the V‐PPase activity under salinity has been obtained; examples for V‐PPase from different plant species have been reported as inhibited, insensitive and activated by salt treatments (Nakamura *et al*., 1992; Colombo & Cerana, 1993; Zingarelli *et al*., 1994; Wang *et al*., 2001; Kabała & Kłobus, 2008; Krebs *et al*., 2010). What could be the reason for these apparent differences? In most studies, the salt effect on the V‐PPase function was quantified only at the level of the hydrolytic enzyme activity. However, it cannot be excluded that proton pumping and PP_i_ hydrolysis of V‐PPases could be very different in intact vacuoles. Furthermore, in mixed vacuole membrane fractions, there is the possibility of contamination by V‐PPase populations from other endomembranes. Here, we directly examined the salt effect on the proton transport capacity of this electroenzyme using the patch‐clamp technique (Fig. 3a). Hence, we provide the first unequivocal evidence that the V‐PPase proton pump activity in *N. benthamiana* mesophyll cells is up‐regulated after salt treatment. Thereby, the enhanced proton pump currents are very likely correlated with a higher abundance of V‐PPase pump proteins in the vacuolar membrane, the result of increased expression of the gene encoding the electroenzyme (Fig. S6d). A salt‐dependent induction of V‐PPase gene expression has previously been reported in wheat and barley (Fukuda *et al*., 2004; Wang *et al*., 2009), suggesting that transcriptional control of the V‐PPase activity in tobacco is not an exception. Salt‐induced stimulation of the NbVHP activity at the post‐translational level, however, is less likely because an increase in vacuolar PP_i_‐triggered proton pump currents was not found in *NbVHP1*‐overexpressing mesophyll cells after salt treatment (Fig. 3a). Under the same scenario with *N. benthamiana*, the V‐ATPase‐dependent pump currents appeared to decrease (Fig. 3b). In this way, the increasing demand for proton pumping and pH homeostasis under salt stress seems to depend mainly on the V‐PPase. In mature leaves and nonstressed conditions, the V‐PPase appears not to play a dominant biological role in plant cells; the loss of V‐PPase function causes only a mild phenotype (Ferjani *et al*., 2011). Our results support the notion that this electroenzyme becomes increasingly important when plants have to cope with stress conditions such as salinity, when additional PMF is required for NHX‐type vacuolar H^+^‐driven Na^+^ sequestration (Apse *et al*., 1999; Yokoi *et al*., 2002; Queirós *et al*., 2009; reviewed in Yamaguchi *et al*., 2013; and controversially discussed in Jiang *et al*., 2010).

In good agreement with the promoting effect on the electrochemical proton gradient, stable overexpression of the homodimericV‐PPase in many transgenic plants from, for example, *A. thaliana*,* Gossypium hirsutum*,* O. sativa*,* Hordeum vulgare*,* Populus trichocarpa*,* Lycopersicon esculentum* and *N. tabacum* has also been shown to improve tolerance to salt as well as to cold and drought stress (Gaxiola *et al*., 2001; Park *et al*., 2005; Gao *et al*., 2006; Zhao *et al*., 2006; Duan *et al*., 2007; Pasapula *et al*., 2011; Schilling *et al*., 2014; Kriegel *et al*., 2015; Yang *et al*., 2015). As a result, stable transgenic V‐PPase plant lines were often characterized by an increased biomass production under stress (Park *et al*., 2005; Gao *et al*., 2006; Pasapula *et al*., 2011; Schilling *et al*., 2014; Yang *et al*., 2015). However, to our knowledge, it has never been reported that V‐PPase overexpression in stable transgenic plant lines reduces plant performance compared with nontransgenic control plants under normal, unstressed growth conditions. We observed that in mature leaves, a transient increase in the endogenous tonoplast‐bound V‐PPases (NbVHP1, NbVHP2) caused a decrease in viability markers such as photosynthetic electron transport and plasma membrane potential (Figs 2b, 5), resulting in cell death, as shown by necrotic patches on the leaf (Fig. 2a). Such a loss of vitality, however, was not found in leaves overexpressing the soluble PPase IPP1. The V‐PPases and the soluble PPase share the ability for PP_i_ hydrolysis, but only the V‐PPases are capable of pumping protons across the tonoplast. Therefore, our data strongly suggest that in transiently V‐PPase‐overloaded mesophyll cells, the fourfold higher proton pump capacity (Fig. 1d) rather than a change in the cellular PP_i_ homoeostasis results in cell damage. Owing to increased transvacuolar membrane proton pumping, NbVHP1‐transformed *N. benthamiana* leaves exhibited a significantly higher acidification of the vacuole than the control (Fig. 4a). This resulted in a stronger pH gradient across the vacuole membrane. By contrast, Duan *et al*. (2007) and Gao *et al*. (2006) observed no changes in the vacuolar pH, although they did find a 40% rise in the hydrolytic V‐PPase activity and an increased biomass of stable AtVHP1/TsVHP‐overexpressing transgenic *N. tabacum* plant lines compared with wild‐type plants. These discrepancies in plant performance and vacuolar pH may be a result of the well‐known phenomena of different expression levels between stable and transient overexpression systems. Unfortunately, the V‐PPase function in these stable V‐PPase‐overexpressing plant lines was rarely quantified; only the PP_i_ hydrolysis activity but not the H^+^ pumping was monitored. For instance, a rise in the hydrolytic V‐PPase enzyme activity of *c*. 12%, 40% and 80% was determined with stable overexpression in *P. trichocarpa*,* N. tabacum* and *O. sativa*, respectively (Gao *et al*., 2006; Zhang *et al*., 2011; Yang *et al*., 2015). In comparison, we obtained a 300% higher V‐PPase proton pump activity with transient overexpression in *N. benthamiana* (Fig. 1d). Thus, plant cells seem to have a tolerance limit for the maximal V‐PPase activity under nonstressed conditions, which is either not detrimental or even beneficial for cell viability and plant growth. This critical threshold may vary during plant development. Moreover, pleiotropic adaption to the higher V‐PPase activity in the stable transgenic plant lines could take place, thus masking or overcoming any otherwise potential negative effect on plant cell performance. However, above the tolerance threshold associated to a physiological context, the increased V‐PPase activity becomes detrimental, as observed with mature leaves under nonstressed conditions (Fig. 2). As a result of the disproportional 300% rise in V‐PPase pump activity in the transient overexpression system, the electrochemical proton gradient across the tonoplast appears to be in excess of the PMFs that can be tolerated under normal growth conditions. Interestingly, the devitalizing effect of transient NbVHP1 overexpression was not observed under salt stress, most likely because of the increased demand for proton‐coupled transport processes at the vacuolar membrane to enable cytosolic sodium ion detoxification.

Our findings clearly indicate that V‐PPase proton pump activity needs to be adapted to the cellular demands; the number of operating pumps needs to be controlled. By this means, both a negative feedback on cell viability by V‐PPase proton‐pump hyperactivity is avoided and – if needed under salt stress – the stress can be relieved to some degree by using the higher PMF for driving secondary active ion‐sequestering transport processes.

## Author contributions

D.G., I.M., M.R.G.R., and R.H designed research; D.G., K.R.K., M.G.P.N., C.L., T.G., J.H., and K.D. performed experiments; D.G., K.R.K., F.B., M.G.P.N., C.L., and T.G. analyzed results; and D.G., K.R.K., F.B., A.F., T.A.C., H.E.N, K.S., R.H., and I.M. wrote the manuscript.

## Supporting information

Please note: Wiley Blackwell are not responsible for the content or functionality of any Supporting Information supplied by the authors. Any queries (other than missing material) should be directed to the *New Phytologist* Central Office.

**Fig. S1** Vectors generated for transient overexpression of pyrophosphatases together with free GFP in *Nicotiana benthamiana* using the agroinfiltration method.
**Fig. S2** Function of AtVHP1 in *Nicotiana benthamiana* mesophyll cells.
**Fig. S3** Comparison of V‐PPase amino acid sequences.
**Fig. S4** Subcellular localization of NbVHP1.
**Fig. S5** Enzyme activity of IPP1.
**Fig. S6** Sodium content, osmolality of apoplastic fluid and NbVHP expression of salt‐treated tobacco leaves.
**Fig. S7** Proton pump activity of V‐PPases overexpressed in *Nicotiana benthamiana* mesophyll cells.
**Fig. S8** pH calibration.
**Methods S1** Generation of mGFP‐NbVHP1‐construct.
**Methods S2** Protein extraction and enzyme activity measurements.
**Methods S3** Quantification of leaf sodium content.
**Methods S4** Apoplast washing.
**Methods S5** Determination of endogenous *NbVHP* transcripts under salt treatment.
**Methods S6** pH calibration with BCECF.
