# Supplementary material for: High V‐PPase activity is beneficial under high salt loads, but detrimental without salinity
Source: New Phytol. 2018 Jun 25;219(4):1421–32. doi: 10.1111/nph.15280 (PMC6099232; doi:10.1111/nph.15280)
Supplement: Supplementary file 1 — Fig. S1 Vectors generated for transient overexpression of pyrophosphatases together with free GFP in Nicotiana benthamiana using the agroinfiltration method. Fig. S2 Function of AtVHP1 in Nicotiana benthamiana mesophyll cells. Fig. S3 Comparison of V‐PPase amino acid sequences. Fig. S4 Subcellular localization of NbVHP1. Fig. S5 Enzyme activity of IPP1. Fig. S6 Sodium content, osmolality of apoplastic fluid and NbVHP expression of salt‐treated tobacco leaves. Fig. S7 Proton pump activity of V‐PPases overexpressed in Nicotiana benthamiana mesophyll cells. Fig. S8 pH calibration. Methods S1 Generation of mGFP‐NbVHP1‐construct. Methods S2 Protein extraction and enzyme activity measurements. Methods S3 Quantification of leaf sodium content. Methods S4 Apoplast washing. Methods S5 Determination of endogenous NbVHP transcripts under salt treatment. Methods S6 pH calibration with BCECF. [file NPH-219-1421-s001.pdf]

*New Phytologist* **Supporting Information**

**Article title**: High V-PPase activity is beneficial under high salt loads, but detrimental without salinity

**Authors**: Dorothea Graus, Kai R. Konrad, Felix Bemm, Meliha Görkem Patir Nebioglu, Christian Lorey, Kerstin Duscha, Tilman Güthoff, Johannes Herrmann, Ali Ferjani, Tracey Ann Cuin, M. Rob G. Roelfsema, Karin Schumacher, H. Ekkehard Neuhaus, Irene Marten, Rainer Hedrich

**Article acceptance date**: 15 May 2018

The following Supporting Information is available for this article:

**Fig. S1** Vectors generated for transient overexpression of pyrophosphatases together with free GFP in *N. benthamiana* using the agroinfiltration method.

**Fig. S2** Function of AtVHP1 in *N. benthamiana* mesophyll cells.

**Fig. S3** Comparison of V-PPase amino acid sequences.

**Fig. S4** Subcellular localization of NbVHP1.

**Fig. S5** Enzyme activity of IPP1.

**Fig. S6** Sodium content, osmolality of apoplastic fluid and NbVHP expression of salt-treated tobacco leaves**.**

**Fig. S7** Proton pump activity of V-PPases overexpressed in *N. benthamiana* mesophyll cells.

**Fig. S8** pH calibration.

**Method S1** Generation of mGFP-NbVHP1 construct.

**Method S2** Protein extraction and enzyme activity measurements.

**Method S3** Quantification of leaf sodium content

**Method S4** Apoplast washing

**Method S5** Determination of endogenous *NbVHP* transcripts under salt treatment

**Method S6** pH calibration with BCECF

**Fig. S1** Vectors generated for transient overexpression of pyrophosphatases together with free GFP in *N. benthamiana* using the agroinfiltration method.

**(a)** Structure of the T-DNA with the gene of interest (NbVHP1, NbVHP2 or IPP1) under the control of the ubiquitin (UBQ) promoter and the coding sequence for the enhanced GFP (eGFP) under the control of the double CaMV35S promoter inserted within the left (LB) and right border (RB). **(b)** Structure of the T-DNA with NbVHP1 inserted by mGFP within cytosolic loop I (between bp 156 and 160). The NbVHP1 coding sequence was under the control of the ubiquitin (UBQ) promoter. RBC-Term = RBC terminator, CaMV35S-Term = CaMV35S terminator.

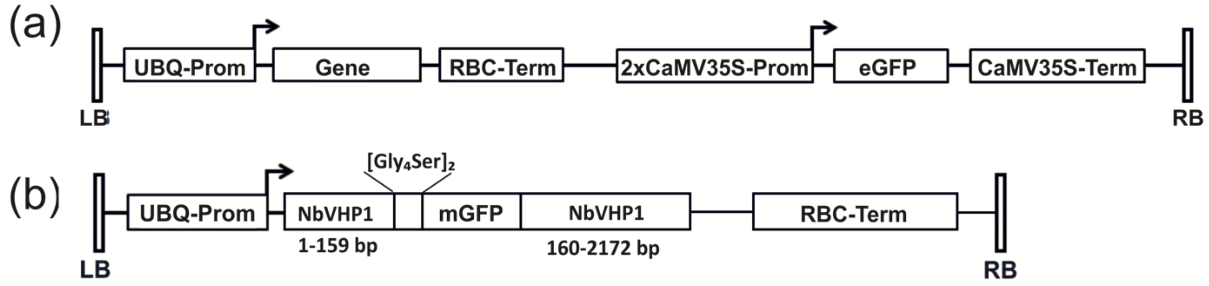

**Fig. S2** Function of AtVHP1 in *N. benthamiana* mesophyll cells.

**(a)** Confocal fluorescence images of a protoplast (left) and a vacuole (right) released from different mesophyll cells overexpressing AVP1 together with free GFP. Red fluorescence is due to chloroplast autofluorescence. Bar scale = 20 µm. **(b)** Representative pyrophosphate-induced current responses of vacuoles released from mesophyll cells overexpressing GFP alone (control) or free GFP together with AtVHP1 after agroinfiltration. Duration of pyrophosphate treatment (150 µM) is indicated by the superimposed grey bars. **(c)** Maximal pyrophosphate-induced changes in current density of vacuoles released from mesophyll cells overexpressing free GFP alone (control, n = 10) or free GFP together with AtVHP1 (n = 20). Pyrophosphate was applied at a concentration of 150 µM. Data represent means ± SE. **(d)** $Ca^{2+}$ dependence of the V-PPase pump activity. Maximal pyrophosphate-induced changes in current density of vacuoles released from mesophyll cells overexpressing free GFP alone (control, n = 3) or free GFP together with AtVHP1 (n = 4). Pyrophosphate (150 µM) was applied to the same vacuole in the presence of either 1 mM $CaCl_2$ or 10 mM EGTA (instead of $CaCl_2$) in the application pipette solution. Otherwise, the composition of the application pipette solution was identical to the bath medium, containing 100 mM KCl, 1 mM $CaCl_2$, 5 mM $MgCl_2$, 10 mM Hepes/Tris pH 7.5. Data represent means ± SE. **(e)** Images from leaves overexpressing free GFP alone (control) or together with AtVHP1 three days after agroinfiltration. **(f)** Maximal pyrophosphate-induced changes in current density of vacuoles released from AtVHP1/GFP-overexpressing mesophyll cells, plotted against the pyrophosphate concentration. Current responses were normalized to those recorded from the same vacuole during application of 150 µM pyrophosphate. Experiments were performed at luminal pH 7.5 (triangle, n = 3-17) or pH 5.5 (circles, n = 3 - 9). Data points (means ± SE) were globally fitted with a Michaelis-Menten equation (solid line).

Asterisks in **(c, d)** indicate significant differences (*$P < 0.05$ and ***$P < 0.001$, Student's t-test) between the given values.

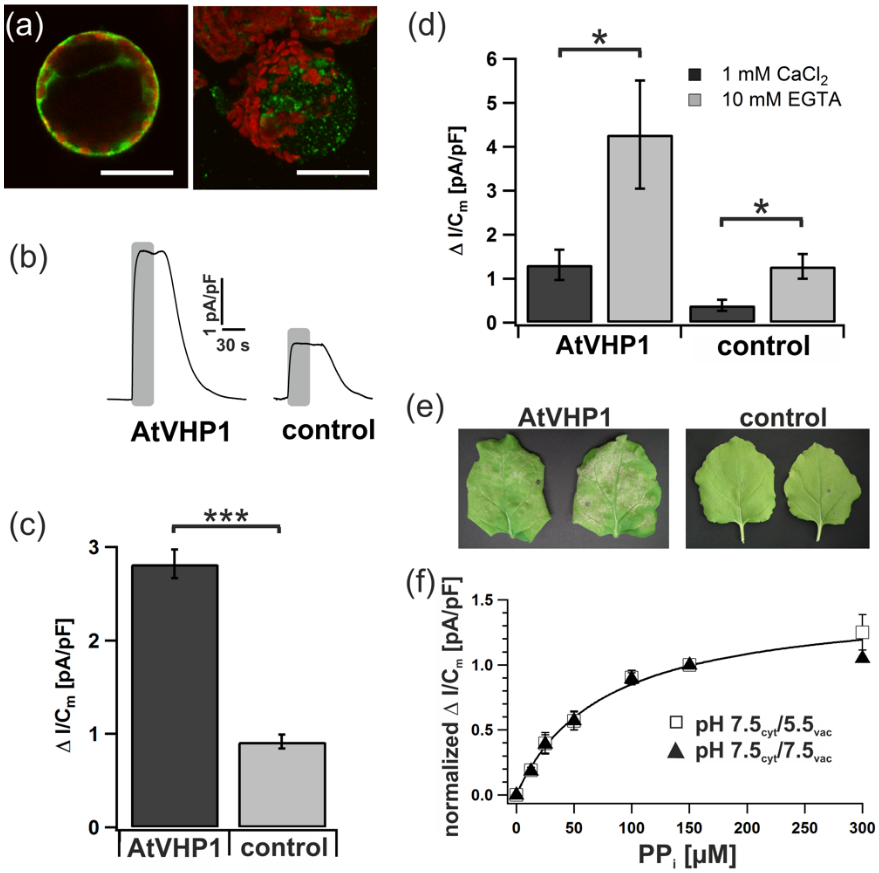

(a)
(b)
1 pA/pF
30 s
AtVHP1
control
(c)
***
Δ I/Cm [pA/pF]
3
2
1
0
AtVHP1
control
(d)
*
6
5
4
3
2
1
0
Δ I/Cm [pA/pF]
1 mM CaCl2
10 mM EGTA
*
AtVHP1
control
(e)
AtVHP1
control
(f)
normalized Δ I/Cm [pA/pF]
1.5
1.0
0.5
0.0
pH 7.5cyt/5.5vac
pH 7.5cyt/7.5vac
0
50
100
150
200
250
300
PPi [µM]

**Fig. S3** Comparison of V-PPase amino acid sequences.

**(a)** Amino acid sequence alignment of V-PPases from *N. benthamiana* (NbVHP1, NbVHP2), *Arabidopsis thaliana* (AtVHP1) and *Vigna radiata* (VrVHP). Percentage agreement of the residues with the consensus sequences is highlighted with a colored background (>80% mid blue, >60% light blue, >40% light grey, <40% white). Conserved amino acids with a proposed role in substrate binding ($K^+$, $Mg^{2+}$, $PP_i$), proton translocation, hydrophobic gate and salt-bridge interactions are highlighted by a green, bright blue, orange and purple background, respectively (Lin *et al.*, 2012). Sequences were aligned using Clustal Omega (CLUSTAL O(1.2.0) multiple sequence alignment; http://www.ebi.ac.uk/Tools/msa/clustalo/) **(b)** Percentage identical in the amino acid sequences of the V-PPases.

(a)

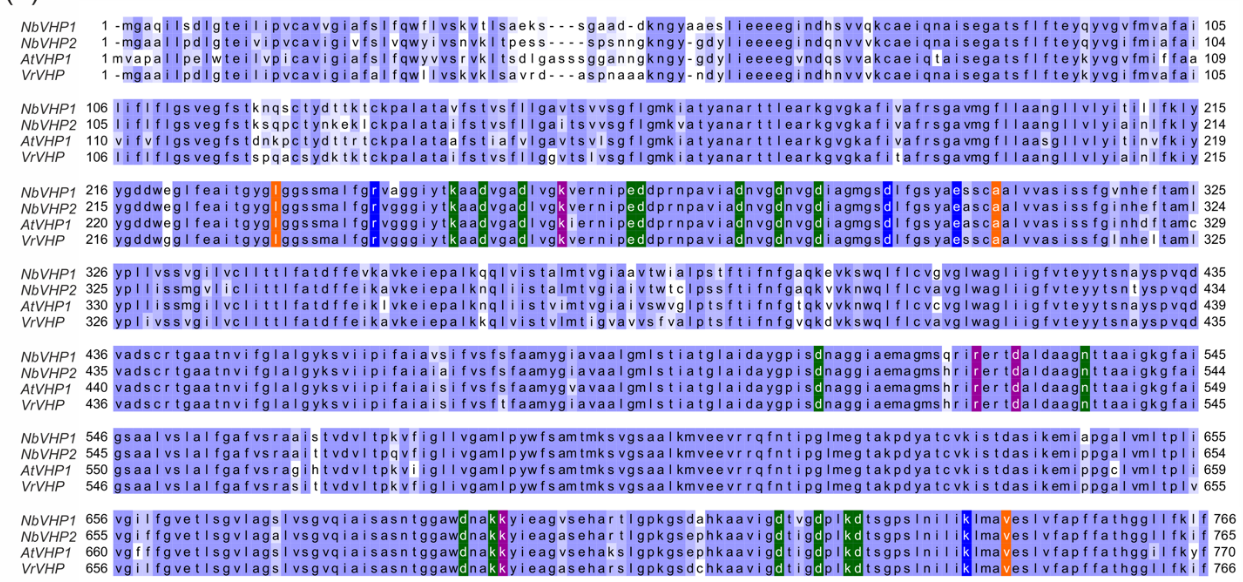

(b)

| | | NbVHP2 |
|---|---|---|
| | NbVHP1 | 91% |
| AtVHP1 | 88% | 90% |
| VrVHP | 91% | 91% |

**Fig. S4** Subcellular localization of NbVHP1.

Confocal fluorescence images of a protoplast **(a)** and a vacuole **(b)** released from different *N. benthamiana* mesophyll cells overexpressing mGFP-labelled NbVHP1. **(a)** The plasma membrane is highlighted with red fluorescence caused by staining with FM4-64. Bar scale = 10 µm. Note that the GFP and FM4-64 fluorescence signals do not co-localize as marked by the arrow. **(b)** Red fluorescence is due to chloroplast autofluorescence. Bar scale = 20 µm.

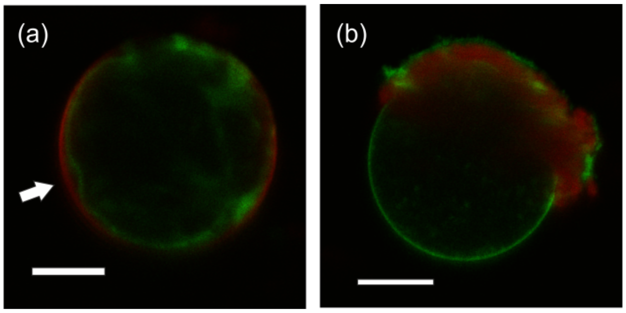

**Fig. S5** Enzyme activity of IPP1.

The hydrolytic pyrophosphate activity of the soluble fraction of *N. benthamiana* leaves overexpressing free GFP alone (control) or together with IPP1 or NbVHP1. Data points (means ± SE, **P < 0.01, Student's t-test) represent three independent experiments.

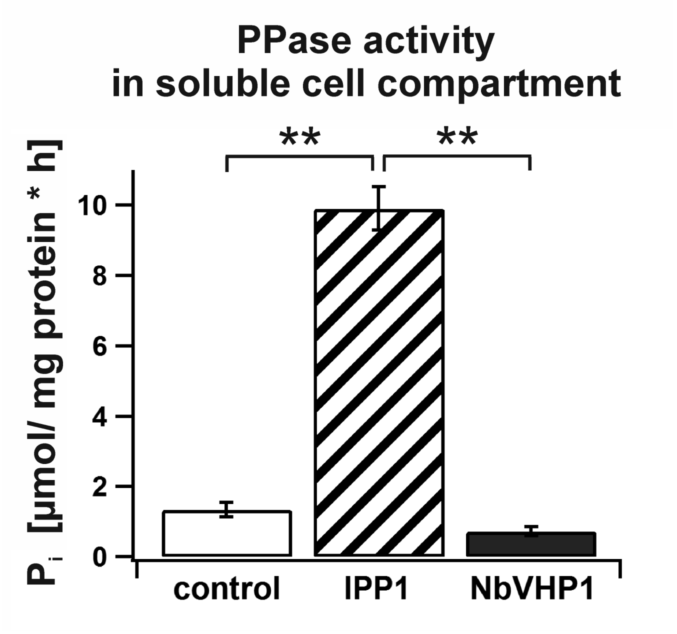

**Fig. S6** Sodium content, osmolality of apoplastic fluid and NbVHP expression of salt-treated tobacco leaves.

**(a)** Sodium content of *N. benthamiana* wild type leaves (DW= Dry weight) without salt treatment (open markers) and at different times after treatment with 200 mM NaCl (closed markers). Plant leaves were directly injected with NaCl (triangle markers) or NaCl was applied in solution to the soil ('poured', circle and square markers). The NaCl content of the 3$^{rd}$ and 4$^{th}$ leaves from the base of the plant were measured separately. Note, that when pots with soil-grown plants were soaked with either water or NaCl, the $Na^+$ content in the third and fourth leaf increased over 48 h to the same $Na^+$ level as observed in $Na^+$ infiltrated leaves, and likewise, then remained stable. **(b)** Sodium content of *N. benthamiana* leaves overexpressing free GFP alone (control) or free GFP together with the indicated pyrophosphatases two days after agroinfiltration with or without 200 mM NaCl. FW = Fresh weight. Significant differences between values at $P < 0.05$ are indicated by different characters (Student's t-test). **(c)** Osmolality of the apoplastic fluid was determined from *N. benthamiana* leaves injected with agromix (light grey) or agromix enriched with 200 mM NaCl (dark grey) after 3-5 h after infiltration. Note that the osmolality of infiltrated pure agromix solution is 25 mosmol kg$^{-1}$. ***$P < 0.001$, Student's t-test. **(d)** Transcript levels of *NbVHP1* and *NbVHP2* in leaves overexpressing free GFP two days after agroinfiltration with or without 200 mM NaCl. *$P < 0.05$, Student's t-test. The transcript levels were normalized to the measured number of *GFP* transcripts.

The number of independent experiments was n = 4 in **(a)** and **(b)**, n = 7 in **(c)** and n = 5 in **(d)** (means ± SE).

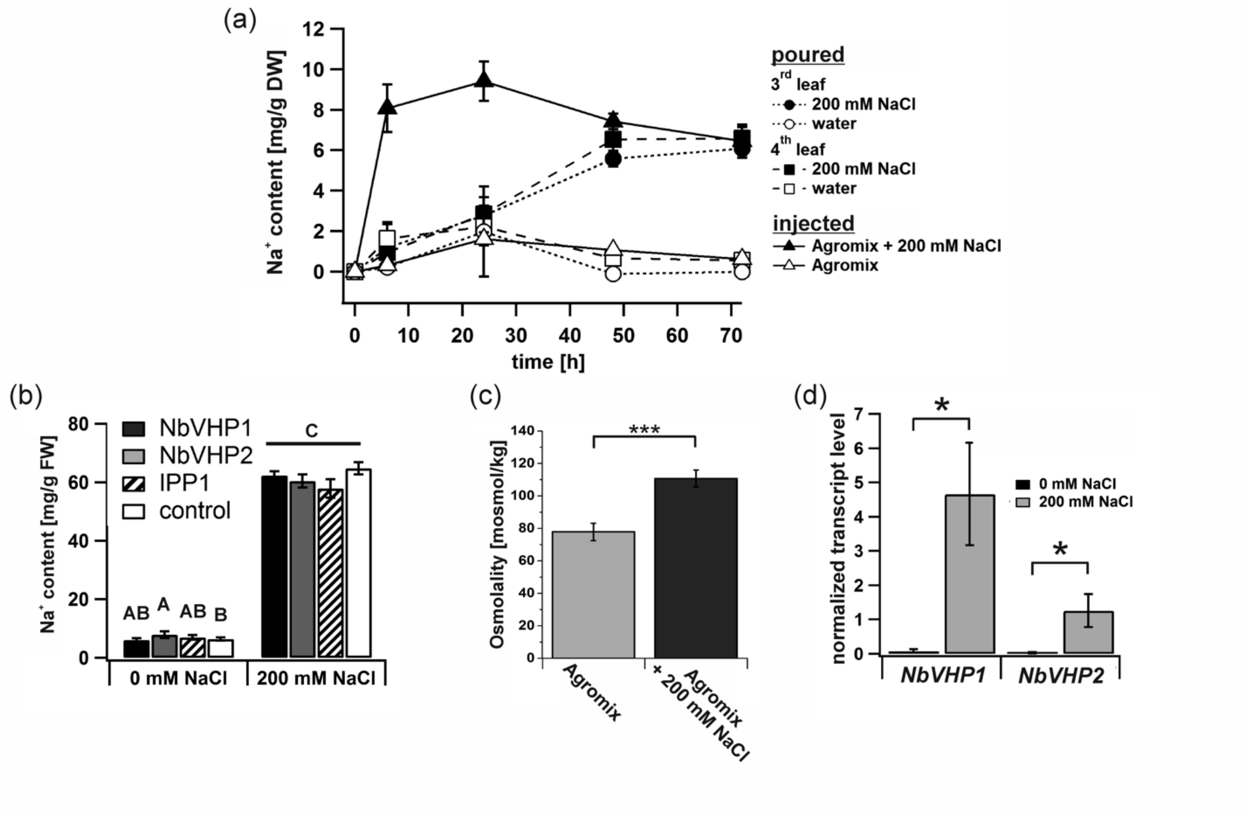
(a)
Na+ content [mg/g DW]
time [h]
poured
3rd leaf
200 mM NaCl
water
4th leaf
200 mM NaCl
water
injected
Agromix + 200 mM NaCl
Agromix
(b)
Na+ content [mg/g FW]
NbVHP1
NbVHP2
IPP1
control
AB
A
AB
B
C
0 mM NaCl
200 mM NaCl
(c)
Osmolality [mosmol/kg]
***
Agromix
Agromix + 200 mM NaCl
(d)
normalized transcript level
*
*
0 mM NaCl
200 mM NaCl
NbVHP1
NbVHP2

**Fig. S7** Proton pump activity of V-PPases overexpressed in *N. benthamiana* mesophyll cells. Maximal pyrophosphate-induced current responses of vacuoles released from mesophyll cells overexpressing free GFP alone (control, n = 10) or free GFP together with either AtVHP1 (n = 22), NbVHP1 (n = 14) or NbVHP2 (n = 4) after agroinfiltration. Pyrophosphate was applied at a concentration of 150 µM. Data points represent means ± SE (***$P < 0.001$, Student's t-test).

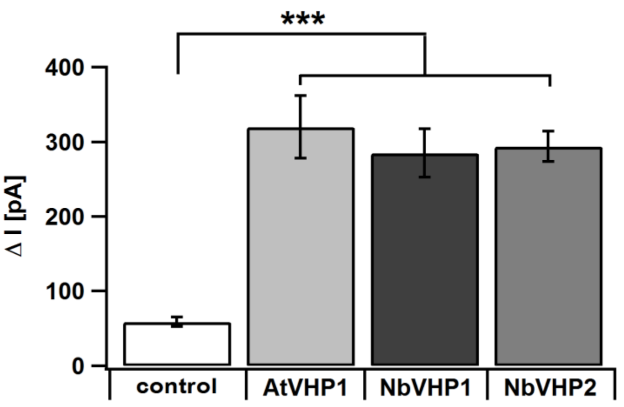


**Fig. S8** pH calibration

BCECF calibration curve generated ex vivo by dissolving the free acid form of the dye (2.5 µM) in a medium containing 1 mM $CaCl_2$, 1 mM MES adjusted to the designated pH with TRIS. Data points (n = 3) were fitted with a linear fit (solid line) to convert ratio values into pH values.

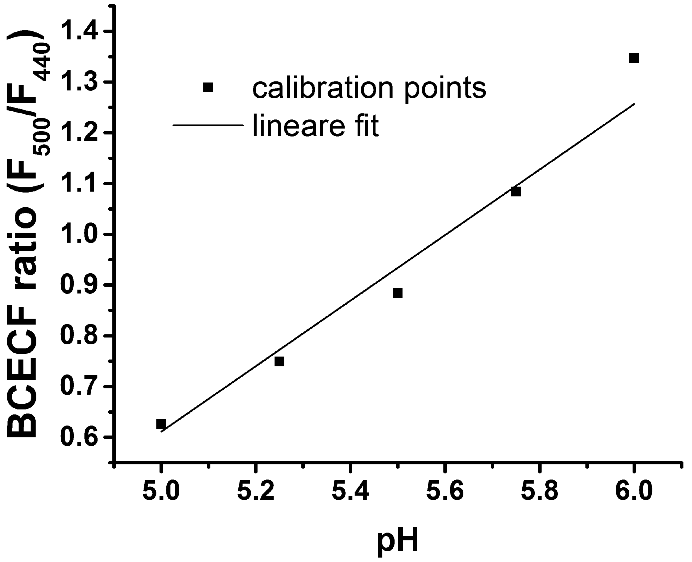

**Method S1** Generation of mGFP-NbVHP1 construct

According to Segami *et al.* (2014), NbVHP1 was labelled with mGFP (monomeric Green Fluorescence Protein) which was inserted into loop 1 exactly between the amino acids Gly53 and Ala54 (Fig. S1b) via USER cloning (Nour-Eldin *et al.*, 2006). Additionally, the following linker sequence was fused to the N-terminus of the mGFP sequence: 5'-GGC GGC GGA GGT TCA GGT GGT GGC GGG TCT -3' (= $[Gly_4Ser]_2$). The following primers were used: mGFPfwd with partial linker-sequence (5'-AGG TTC AGG TGG UGG CGG GTC TGC TGT GAG CAA GGG CGA GG-3'), mGFPrev (5'-AGA TGC CTT GUA CAG CTC GTC CAT GCC GTG A-3'); NbVHP1-L1rev with partial linker sequence (5'-ACC ACC TGA ACC UCC GCC GCC TGA CTT TTC AGC ACT GAG CGT C-3'); NbVHP1_L1fwd (5'-ACA AGG CAT CTG GAG CAG CAG ACG ATA AGA ATG G-3'). Using the USER cloning system, the NbVHP1-mGFP construct was transferred into the pCambia2200 vector equipped with UBQ promotor and RBC terminator.

For staining the plasma membrane, isolated protoplasts were briefly incubated with FM$^{TM}$4-64 (10 µM) (Thermo Fisher Scientific). The FM4-64 dye was excited with 514 nm and the emission detected at 625-690 nm.

**Method S2** Protein extraction and enzyme activity measurements

Two days after agroinfiltration, tobacco leaves were harvested, immediately frozen in liquid nitrogen and stored at -80 °C until microsomal membranes and soluble protein fractions were prepared as described previously (Krebs *et al.*, 2010), with minor modifications. Tissue was homogenized with 2 ml homogenization buffer per g fresh weight (350 mM sucrose, 70 mM Tris-HCl pH 8.0, 10 % (v/v) glycerol, 3 mM $Na_2EDTA$, 0.15 % (w/v) BSA, 1.5 % (w/v) PVP-40, 4 mM DTT, and 1 x complete protease inhibitor mixture (Roche)). The homogenate was filtered through two layers of Miracloth (Calbiochem) and centrifuged at 15,000 *g* for 15 min at 4 °C. The supernatant was filtered again through Miracloth, then centrifuged at 100,000 *g* for 1 h at 4 °C. To determine enzyme activity of the soluble protein fraction, 200 µl of the supernatant was retained and the remaining supernatant was carefully removed and discarded. The pellet (microsomal

membranes) was re-suspended in 350 mM sucrose, 10 mM Tris-Mes pH 7.0, 2 mM DTT and 1 × complete protease inhibitor mixture.

To determine the hydrolytic PPase enzyme activity, the $P_i$ release was determined colorimetrically as described previously (Krebs *et al.*, 2010), with minor modifications. Soluble protein fraction (10 µg) were incubated for 20 min at 28 °C. Reactions were terminated by adding 40 mM citric acid. 10 µg BSA was used for the blank value. The PPase reaction medium contained 25 mM Tris-MES pH 7.5, 2 mM $MgSO_4 \times 7\ H_2O$, 0.1 mM $Na_2MoO_4$, 0.1 % Brij 58, and 0.2 mM $K_4P_2O_7$. PPase activity was calculated as the difference measured in the presence and absence of 50 mM KCl.

**Method S3** Quantification of leaf sodium content

*N. benthamiana* leaves infiltrated only with 200 mM NaCl were harvested at different times after salt treatment, dried at 60 °C to constant weight, pulverized and homogenized. After the exact amount of the samples (usually 10 - 20 mg) were precisely weighed in a quartz digestion vessel, the leaf tissue was treated with 1 ml of nitric acid (65%, suprapur, Merck KGaA, Darmstadt) and digested at 180 °C for 10 hours inside a Teflon pressure vessel. After cooling, samples were diluted 1:20 with nanopure water. The sodium content was determined using a flame atomic absorption spectrometer (AAnalyst 400, PerkinElmer) three times and averaged.

For tobacco leaves infiltrated with a NaCl-free or 200 mM NaCl-containing agrobacterium suspension, leaves were collected two days after infiltration, immediately frozen in liquid nitrogen and stored at -80 °C until use. After re-suspension in double-distilled water the plant leaf material was subjected to complete hydrolysis using a temperature step gradient with a maximum temperature of 210 °C. Hydrolysis was carried in a MLS-Ethos microwave oven (http://www.mls-mikrowellen.de/) with 5 ml $HNO_3$ (60% v/v), 2 ml $H_2O_2$ (30% v/v), and the preparation was diluted to a final volume of 12 ml with double-distilled water. Quantification was performed by inductively coupled plasma/optical emission spectrometry, on an iCAP 6300 DUO apparatus (Thermo-Fischer). Sodium was detected and quantified at 589.5 nm (Krebs *et al.*, 2010; Müller *et al.*, 2014).

**Method S4** Apoplast washing

Agromix solution with or without 200 mM NaCl was infiltrated into the apoplast of wild type *N. benthamiana* leaves through the open stomata via a syringe. After recovery from infiltration (about 3-5 h) leaves were harvested and the surface washed and infiltrated a second time with distilled water. The leaf apoplast fluid was then extracted by centrifugation as described by O'Leary *et al.* (2014), and its osmolality measured with an osmometer (Vapor Pressure Osmometer 5520, Wescor, Vapro).

**Method S5** Determination of endogenous *NbVHP* transcripts under salt treatment

*N. benthamiana* leaves were harvested two days after agroinfiltration with the appropriate constructs for overexpression of GFP alone (control) in the presence or absence of 200 mM NaCl. Plant material was immediately frozen in liquid nitrogen, crushed with a pestle and mortar and stored at -80°C until use. Total RNA of the leaf was isolated using Plant RNA Kit R6827-02 (OMEGA, www.omegabiotek.com). Each of the collected RNAs was treated with recombinant DNase (Thermo Scientific) to remove any genomic DNA contamination. Generation of cDNA and Quantitative real-time PCR (pPCR) were done like described in the main text. Transcription data were normalized to the coexpressed GFP molecules using standard curves calculated for the individual PCR products. GFPfwd (5′-CCT GAA GTT CAT CTG CAC CA-3′) and GFPrev (5′-TGC TCA GGT AGT GGT TGT CG-3′) (Gaddam *et al.*, 2013).

**Method S6** pH calibration with BCECF

A BCECF calibration curve was generated *ex vivo* with exactly the same imaging parameters and setup used for the *in vivo* measurements described in the main text by dissolving the free acid form of the dye (2.5 µM) in a medium containing 1 mM $CaCl_2$, 1 mM MES adjusted to the designated pH with TRIS (Fig. S8). The generated calibration curve (n = 3) was used to convert the ratio values into pH-values.

# References

**Gaddam D, Stevens N, Hollien J**. **2013**. Comparison of mRNA localization and regulation during endoplasmic reticulum stress in Drosophila cells. *Molecular Biology of the Cell* **24**: 14–20.

**Krebs M, Beyhl D, Görlich E, Al-Rasheid KAS, Marten I, Stierhof Y-D, Hedrich R, Schumacher K**. **2010**. Arabidopsis V-ATPase activity at the tonoplast is required for efficient nutrient storage but not for sodium accumulation. *Proceedings of the National Academy of Sciences of the United States of America* **107**: 3251–3256.

**Lin S-M, Tsai J-Y, Hsiao C-D, Huang Y-T, Chiu C-L, Liu M-H, Tung J-Y, Liu T-H, Pan R-L, Sun Y-J**. **2012**. Crystal structure of a membrane-embedded $H^+$-translocating pyrophosphatase. *Nature* **484**: 399–403.

**Müller M, Kunz H-H, Schroeder JI, Kemp G, Young HS, Neuhaus HE**. **2014**. Decreased capacity for sodium export out of Arabidopsis chloroplasts impairs salt tolerance, photosynthesis and plant performance. *The Plant Journal* **78**: 646–658.

**Nour-Eldin HH, Hansen BG, Nørholm MHH, Jensen JK, Halkier BA**. **2006**. Advancing uracil-excision based cloning towards an ideal technique for cloning PCR fragments. *Nucleic Acids Research* **34**: e122.

**O'Leary BM, Rico A, McCraw S, Fones HN, Preston GM**. **2014**. The infiltration-centrifugation technique for extraction of apoplastic fluid from plant leaves using phaseolus vulgaris as an example. *Journal of Visualized Experiments : JoVE* **19**:e52113.

**Segami S, Makino S, Miyake A, Asaoka M, Maeshima M**. **2014**. Dynamics of vacuoles and $H^+$-pyrophosphatase visualized by monomeric green fluorescent protein in Arabidopsis: artifactual bulbs and native intravacuolar spherical structures. *The Plant Cell* **26**: 3416–3434.
